# Supplementary material for: Association of Night Eating Habits with Health-Related Quality of Life (HRQoL) in University Students
Source: Healthcare (Basel). 2022 Mar 28;10(4):640. doi: 10.3390/healthcare10040640 (PMC9031917; doi:10.3390/healthcare10040640)
Supplement: Supplementary file 1 [file healthcare-10-00640-s001.zip › healthcare-1649098_supp_eng.pdf]

Questionnaire on late-night snacking habits

▪ This questionnaire is about your night eating habits. Please tick (✔) the appropriate responses. *Night eating: A meal or snack eaten late at night after dinner

[Frequency of night eating]

1. How often do you eat late-night snacks?

① Less than once a week (Go to question 2)

② Once or twice a week

③ 3 to 4 times a week

④ More than 5 times a week

[Reason for night eating]

1-1. What is the main reason for your late-night snacking?

① Because I feel hungry

② Because people around me hand me snacks

③ To relieve stress

④ Food-related TV programs or commercials make me want to eat

⑤ Force of habit

⑥ Others

[Quantity of night eating]

2. How much do you usually eat when you have late-night snacks?

① Very little

② Little

③ Moderate amount

④ Quite a bit

⑤A lot

[Factors influencing night eating]

3. Who influences you the most in choosing a late-night snack?

① Parents

② Siblings

③ Friends

④ Myself

⑤ Media (TV, radio, SNS, apps, etc.)

4. Who provides you late-night snacks?

①My parents make it for me

②My parents buy it for me

③I make them myself

④I buy them myself

⑤ Others

[Night eating with companion(s)]

5. With whom do you usually eat late-night snacks?

① I eat alone

② With my parents

③ With my siblings

④ With the whole family

⑤ With my friends

[Place of night eating]

6. Where do you usually eat your late-night snacks?

① At home

② At convenience stores or supermarkets

③ On the street or at street stalls

④ At fast-food restaurants

⑤ At school

⑥ At a restaurant

[Cessation point of night eating]

7. When do you stop late-night snacking?

① When I finish eating all the food

② When I feel full

③ When I feel that I've not had enough

④ When everyone around me has finished snacking

[Night eating time]

8. When do you usually eat late-night snacks?

① Before 8:00 p.m.

② 8:00 p.m. to 10:00 p.m.

③ 10:00 pm to 12:00 a.m.

③ 12:00 a.m. to 2:00 a.m.

④ After 2:00 a.m.

[Going to bed after night eating]

9. How long after night eating do you usually go to bed?

① Within than 30 minutes

② 30 minutes to less than 1 hour

③ 1 hour to less than 2 hours

④ After 2 hours

[Accompanying drinks when night eating]

10. What do you usually drink when eating late-night snacks? (Multiple choices are possible)

① Soft drinks (coke, soda, etc.)

② Alcoholic beverages

③ Juice (fruit juice, vegetable juice, etc.)

④ Water

⑤ Tea (green tea, coffee, barley tea, herbal tea, etc.)

⑥ Ion drink

⑦ Dairy products (milk, coffee with milk)

⑧ None

[Drink-related questions]

11. How often do you drink a beverage when having snacks, as responded to in question 10?

① every time

② 5 to 6 times

③ 3 to 4 times

④ Once or twice

12. What do you drink most often?

① Wine

② Beer

③ Soju

④ Makgeolli

⑤ Western liquor

⑥ Cocktail

⑦ Bomb cocktail

⑧ None

13. How often do you drink alcohol?

① I don't drink (Go to question 16)

② Once or twice a week

③ 2 to 3 times a week

④ 3 to 4 times a week

⑤ 4 to 5 times a week

⑥ every day

14. Where do you usually drink alcohol?

① Near my home

② Near school

③ Downtown

④ At home

15. How much alcohol do you drink when you eat a late-night snack?

(Write the amount of alcohol you drink most often)

① ( )bottle(s) of soju

② ( )bottle(s)or ( )can(s) of beer

③ ( )bottle(s) of makgeolli

④ ( )bottle(s) of Western liquor

⑤ ( )bottle(s) of wine

⑥ ( )glass(es) of bomb cocktail

⑦ ( )glass(es) of cocktail

[Preferences by type of night eating]

16. The following are the preferences within each type of late-night snack. Please mark your preferred type and your level of liking toward each type in the appropriate box.

Category	Food type	Love it	Like it	So-so	Dislike it	Hate it
Meat	Pork hocks					
	Bossam					
	Grilled pork belly, beef, etc.					
Noodles	Ramen, Cup					

	noodles					
	Spaghetti					
	Noodle					
	Jjajangmyeon, Jjamppong					
	Cold noodles					
Casual food	Kimbab					
	Tteokbokki					
	Mandu					
	Sundae					
Fast food	Hamburgers					
	Pizza					
	Chicken					
Bread and Confectionery	Bread, cakes					
	Sandwiches					
	Biscuits, snacks					
	Chocolate, candies					
Cereal	Cereal					
Fruits	Fresh fruit					
	Dried fruit					
Milk and dairy products	Milk					
	Dairy products (Yogurt, cheese)					
Beverages	Coffee					
	Tea					

	Soft drink					
	Ion drink					
	Fruit juice					
Frozen desserts	Ice cream					
Nuts	Nuts					

[Preferred taste]

17. Do you have any taste preference when choosing a late-night snack?

① Sweet ② Salty ③ Spicy ④ Light ⑤ Oily

[Criteria for choosing a late-night snack]

18. What consideration do you give to each of the following when you choose a late-night snack?

No.	Category	Very important	Important	Moderate	A little important	No importance
1	Taste					
2	Price					
3	Quantity of food					
4	Calorie					
5	Hygiene					
6	Nutrition					
7	Food source					
8	Brand					
9	Advertisement of the product					
10	Popularity					

11	Reliability					
